# A Novel Three-miRNA Signature Identified Using Bioinformatics Predicts Survival in Esophageal Carcinoma

**DOI:** 10.1155/2020/5973082

**Published:** 2020-02-10

**Authors:** KunZhe Wu, ChunDong Zhang, Cheng Zhang, DongQiu Dai

**Affiliations:** Department of Gastrointestinal Surgery, The Fourth Affiliated Hospital of China Medical University, Shenyang 110032, China

## Abstract

**Objective:**

We identified differentially expressed microRNAs (DEMs) between esophageal carcinoma (ESCA) tissues and normal esophageal tissues. We then constructed a novel three-miRNA signature to predict the prognosis of ESCA patients using bioinformatics analysis. *Materials and Methods*. We combined two microarray profiling datasets from the Gene Expression Omnibus (GEO) database and RNA-seq datasets from the *Cancer* Genome Atlas (TCGA) database to analyze DEMs in ESCA. The clinical data from 168 ESCA patients were selected from the TCGA database to assess the prognostic role of the DEMs. The TargetScan, miRDB, miRWalk, and DIANA websites were used to predict the miRNA target genes. Functional enrichment analysis was conducted using the Database for Annotation, Visualization, and Integrated Discovery (David), and protein-protein interaction (PPI) networks were obtained using the Search Tool for the Retrieval of Interacting Genes database (STRING).

**Results:**

With cut-off criteria of *P* < 0.05 and |log2FC| > 1.0, 33 overlapping DEMs, including 27 upregulated and 6 downregulated miRNAs, were identified from GEO microarray datasets and TCGA RNA-seq count datasets. The Kaplan–Meier survival analysis indicated that a three-miRNA signature (miR-1301-3p, miR-431-5p, and miR-769-5p) was significantly associated with the overall survival of ESCA patients. The results of univariate and multivariate Cox regression analysis showed that the three-miRNA signature was a potential prognostic factor in ESCA. Furthermore, the gene functional enrichment analysis revealed that the target genes of the three miRNAs participate in various cancer-related pathways, including viral carcinogenesis, forkhead box O (FoxO), vascular endothelial growth factor (VEGF), human epidermal growth factor receptor 2 (ErbB2), and mammalian target of rapamycin (mTOR) signaling pathways. In the PPI network, three target genes (*MAPK1*, *RB1*, and *CLTC*) with a high degree of connectivity were selected as hub genes.

**Conclusions:**

Our results revealed that a three-miRNA signature (miR-1301-3p, miR-431-5p, and miR-769-5p) is a potential novel prognostic biomarker for ESCA.

## 1. Introduction

Esophageal carcinoma (ESCA), including esophageal adenocarcinoma and esophageal squamous cell carcinoma, is characterized by aggressive malignant tumor formation [1]. It is the eighth most common type of cancer and the sixth most frequent cause of cancer-related death globally [2]. There were an estimated 572,000 new ESCA diagnoses worldwide in 2018, which accounted for 3.2% of all malignancies [3]. The overall 5-year survival rate was only 15%–25% [4]. Thus, there is an urgent need to discover novel effective prognostic biomarkers for assisting in early detection and improved treatment of ESCA.

MicroRNA (miRNA) is one type of noncoding RNA, approximately 19–25 nucleotides in length, which regulates cellular proliferation, migration, and invasion by modifying gene expression [5]. MiRNAs can regulate target genes by binding to the 3′-untranslated region to repress translation or regulate degradation [6]. It has been reported that dysregulated miRNAs could be a potential biomarker for tumor diagnosis [7]. Therefore, significantly differentially expressed microRNAs (DEMs) in ESCA could be effective biomarkers for early diagnosis, therapeutic strategy selection, and prognosis of ESCA.

Previous studies have reported that a number of DEMs are associated with ESCA prognosis [8–10]. However, these studies had limitations including clinical heterogeneity, sample insufficiency, and differences in various data processing methods. A large sample size with detailed clinical features is important for reliable survival prediction in patients with ESCA. The Gene Expression Omnibus (GEO) database provides an invaluable resource for gene expression data and other functional genomics data [11]. The *Cancer* Genome Atlas (TCGA) project contains sequencing data on more than 11,000 miRNAs as well as clinical information from cancer patients [12].

In this study, we detected DEMs between ESCA tissues and normal tissues through the integration of two microarray profiling datasets from the GEO database and RNA-seq datasets from the TCGA database. The prognostic value of the identified DEMs was evaluated by using clinical features downloaded from the TCGA database, and a novel three-miRNA signature was built to determine the prognosis of ESCA patients. Functional enrichment analysis was performed and protein-protein interaction (PPI) networks were obtained to elucidate interactions and identify characteristics of the potential common target gene of the three miRNAs.

## 2. Materials and Methods

### 2.1. Data Mining of GEO and Identification of DEMs

The microarray profiling datasets of GSE43732 (including 119 ESC samples and 119 adjacent noncancerous samples) and GSE55856 (including 108 ESC samples and 108 adjacent noncancerous samples) with large sample sizes were obtained from the GEO database. In addition, we downloaded ESCA miRNA-Seq with associated clinical data from the TCGA database. A total of 181 samples consisting of 168 ESCA and 13 normal esophageal samples were analyzed to identify DEMs. After normalization and log2 transformation of the data from the original GEO datasets, the DEMs between ESCA tissue samples and normal esophageal tissue samples were analyzed by the limma package of *R* software [13]. The EdgeR package of *R* was used to explore DEMs for the RNA-seq count datasets from the TCGA database [14]. All miRNAs meeting the criteria of *P* < 0.05 and |log2FoldChange (log2FC)| > 1.0 were considered as DEMs. Volcano plots and hierarchical clustering heatmaps (the top 30 DEMs) of the microarray profiling datasets from GEO and the RNA-seq count datasets from the TCGA database were drawn using *R* software.

### 2.2. DEMs and Overall Survival (OS) of Patients with ESCA

The RNA-seq data and corresponding clinical information for ESCA were downloaded from the TCGA database. The following inclusion criteria were used: (1) samples with necessary clinical data for analysis, (2) patients without preoperative radiotherapy and chemotherapy, and (3) OS time exceeding two weeks. A total of 181 samples were selected in this study, including 13 normal tissues and 168 ESCA tissues. The prognostic value of the DEMs was examined by the Kaplan–Meier method and the log-rank test. Subsequently, the statistically significant prognosis-related miRNAs were ranked by median expression level, and each ESCA patient was scored in accordance with a high or low level of expression, and a risk grade was defined by the total scores. Then, ESCA patients were sorted into a high-risk group or low-risk group according to the risk-score rank. The differences in patients' survival between the two groups were evaluated by using the Kaplan–Meier survival method. Finally, a prognostic three-miRNA signature was established by integrating the expression profiles of three miRNAs and corresponding estimated regression coefficient. Furthermore, the expression levels of the three DEMs were analyzed with RNA sequencing expression data of normal control tissue samples and ESCA tissue samples in TCGA database.

### 2.3. Functional Enrichment Analysis

The common target genes of the three miRNAs were predicted using the miRDB (http://www.mirdb.org/), TargetScan (http://www.targetscan.org/), miRWalk (http://zmf.umm.uniheidelberg.de/apps/zmf/mirwalk2/), and DIANA (http://www.microrna.gr/microT-CDS) online bioinformatics tools. Venn diagrams were used to obtain overlapping target genes identified by these four bioinformatics tools. GO annotation (http://www.geneontology.org) and KEGG signaling pathway were set up using the Database for Annotation, Visualization, and Integrated Discovery (David) (https://david.ncifcrf.gov/) bioinformatics tool [15].

### 2.4. Hub Nodes in the PPI Network

The Search Tool for the Retrieval of Interacting Genes (STRING) database (http://www.string-db.org) and Cytoscape (version 3.4.0) were used to construct a PPI network of common target genes. The hub nodes in the PPI network were investigated topologically, and PPI pairs with a combined score of more than 0.4 were selected to construct the PPI network. The crucial genes were identified by eigenvector centrality (EGC), degree centrality (DC), and closeness centrality (CC), which were calculated using the Cytoscape plugin CytoNCA [16].

### 2.5. Statistical Analyses

All statistical analyses were performed using SPSS 20.0 (SPSS Inc., Chicago, IL, USA) and Prism 7.0 (GraphPad, Inc., La Jolla, CA, USA) software. The comparisons of DEM expression and clinical characteristics between the two groups were performed using the chi-square test and Student's *t*-tests. Univariate and multivariate Cox analyses were performed to detect the relationship between prognostic features and DEMs. *P* values < 0.05 were considered statistically significant.

## 3. Results

### 3.1. Identification of DEMs in ESCA

**Figure 1:**
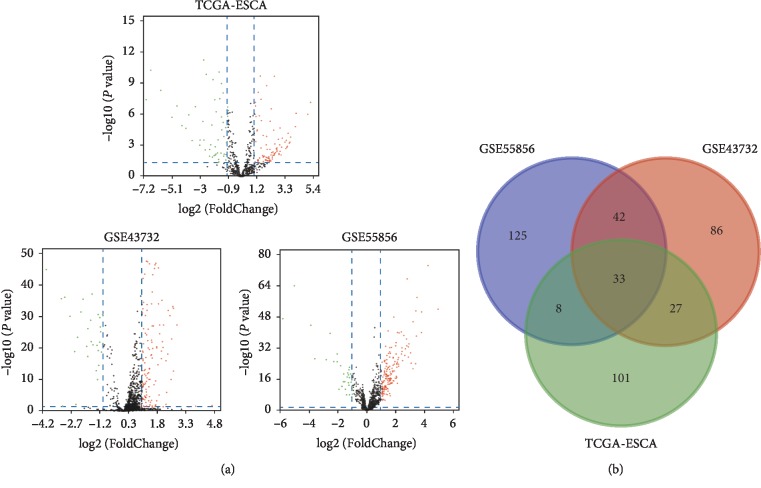
Identification of DEGs among TCGA and GEO datasets of ESCA. (a) Volcano plot of miRNA expression profile data between ESCA tissues and normal tissues in each dataset. Red dots: significantly upregulated genes in ESCA; Green dots: significantly downregulated miRNAs in ESCA; Black dots: nondifferentially expressed miRNAs. *P* < 0.05 and |log2 FC| > 1.0 were considered as significant. (b) The Venn diagram showing the overlapped miRNAs in three datasets. ESCA and esophageal carcinoma.

**Figure 2:**
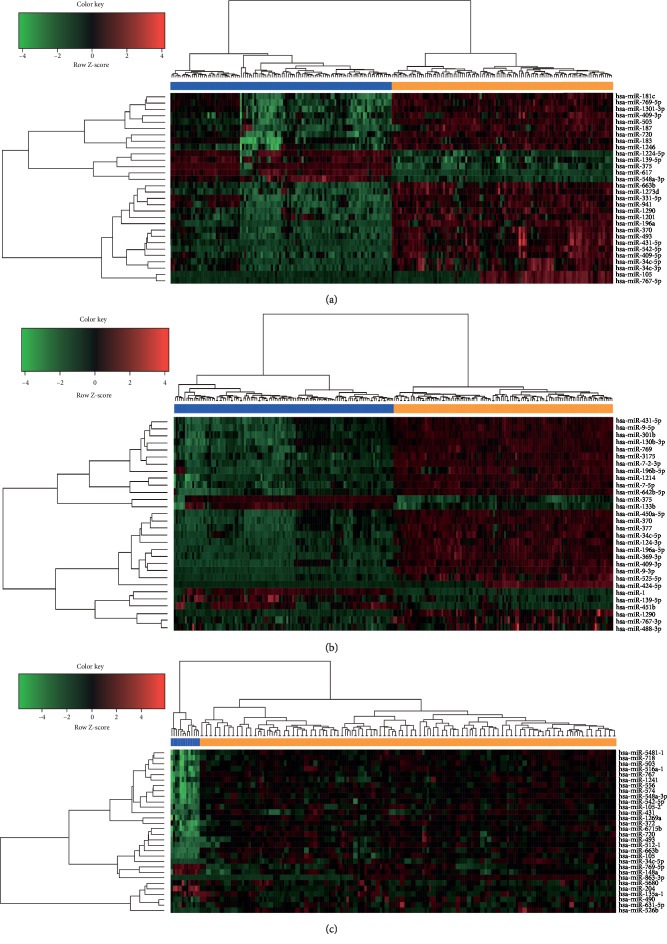
Hierarchical clustering heatmap of top 30 differentially expressed miRNAs. (a) GSE43732 data, (b) GSE55856 data, and (c) TCGA RNA-seq data. Each row represents the expression level of a miRNA, and each column represents a sample: orange for esophageal cancer and blue for normal.

Using the cut-off criteria of *P* < 0.05 and |log2FC| > 1.0, we obtained 188 DEMs (including 158 upregulated and 30 downregulated miRNAs) from GSE43732, 208 DEMs (including 175 upregulated and 33 downregulated miRNAs) from GSE55856, and 169 DEMs (including 117 upregulated and 52 downregulated miRNAs) from TCGA RNA-seq datasets. The downregulated miRNAs and upregulated miRNAs in microarray profiling datasets and the RNA-seq count dataset are displayed in the volcano plots in Figure 1(a). Moreover, a total of 33 DEMs were found to overlap in the GEO microarray datasets and TCGA RNA-seq count datasets, of which 27 were significantly upregulated and 6 were downregulated, as shown in a Venn diagram (Supplementary [Supplementary-material supplementary-material-1], Figure 1(b)). The hierarchical cluster heatmaps (the top 30 DEMs) of each GEO microarray dataset and TCGA dataset are shown in Figure 2.

### 3.2. Association between DEMs and OS of ESCA Patients

**Figure 3:**
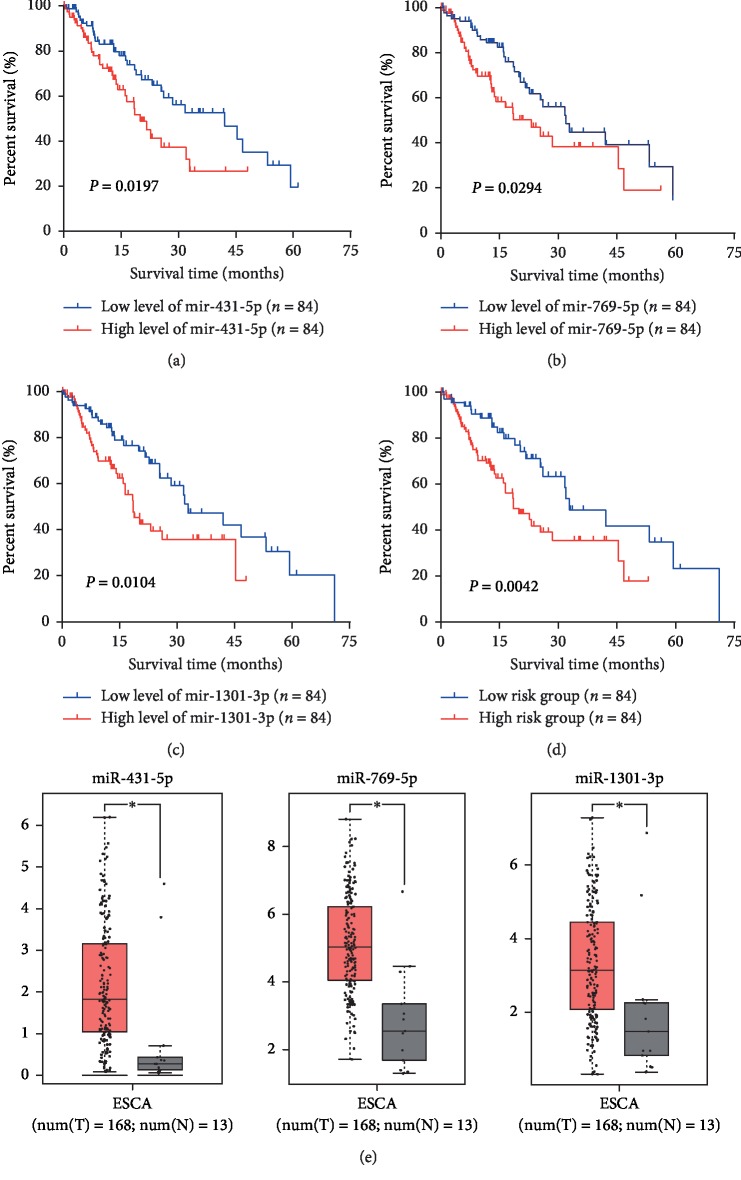
Kaplan–Meier survival curves and box plots. Kaplan–Meier survival curves for three miRNAs (a) miR-431-5p, (b) miR-769-5p, and (c) miR-1301-3p associated with the overall survival of patients with ESCA. (d) Kaplan–Meier survival curve for the three-miRNA signature in patients with ESCA. (e) Comparisons of the expression levels of the three miRNAs between ESCA and non-ESCA tissues based on TCGA data. The red ^*∗*^*P* < 0.05; ESCA, esophageal carcinoma.

To explore the prognostic roles of each DEM in ESCA patients, data from 168 ESCA tumor samples and 13 normal tissue samples were downloaded from the TCGA database. Associated clinical characteristics included sex, age, pathologic stage, T stage, lymph node status, metastasis, and histological stage (Table 1). Subsequently, the Kaplan–Meier survival analysis was performed to identify the relationship between each DEM and OS of ESCA patients (Supplementary [Supplementary-material supplementary-material-1]). We found that three DEMs (miR-1301-3p, miR-431-5p, and miR-769-5p) had a negative association with OS (Figures 3(a)–3(c)). Then, clinical and pathological analyses indicated that miR-431-5p was significantly associated with the pathologic stage (*P*=0.048), miR-1301-3p was significantly associated with the T stage (*P*=0.014), and miR-769-5p and miR-1301-3p were both significantly associated with histologic grade (*P* < 0.05) (Table 2).

### 3.3. The Expression and Prognostic Value of the Three DMEs

Furthermore, TCGA data were used to validate the expression of the three DEMs. We found that miR-1301-3p, miR-431-5p, and miR-769-5p were upregulated in TCGA databases (Figure 3(e)). Based on the risk grade results, all 168 ESCA patients were classified into a high- or low-risk group. The Kaplan–Meier survival analysis indicated that lower expression of the three-miRNA signature was correlated with a higher survival rate of ESCA patients (*P*=0.0042) (Figure 3(d)). In addition, we performed univariate and multivariate Cox regression analyses to examine the prognostic role of the three-miRNA signature (high-risk vs. low-risk) according to clinical features. The results of univariate analysis indicated that the pathological stage (hazard ratio [HR] = 2.065, *P* < 0.001), *T* stage (HR=1.474, *P*=0.023), lymph node status (HR=1.395, *P*=0.003), distant metastasis (HR=2.163, *P*=0.006), and expression of the three DEMs (HR=3.234, *P*=0.004) were significantly associated with the prognosis of ESCA patients. In a multivariate analysis, pathologic stage (HR=1.995, *P*=0.002) and the three-miRNA signature (HR=3.442, *P*=0.004) were shown to be potential independent factors in predicting the prognosis of ESCA patients (Table 3).

### 3.4. Functional Enrichment Analysis

**Figure 4:**
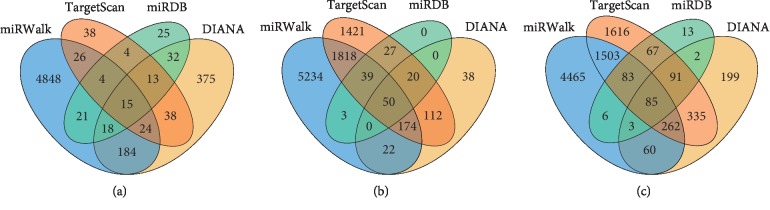
Target gene prediction of each differentially expressed miRNA. The overlapping target genes were predicted using the miRDB, TargetScan, miRWalk, and DIANA online analysis tools. (a) miRNA-431-5p; (b) miRNA-769-5p; and (c) miR-1301-3p.

**Figure 5:**
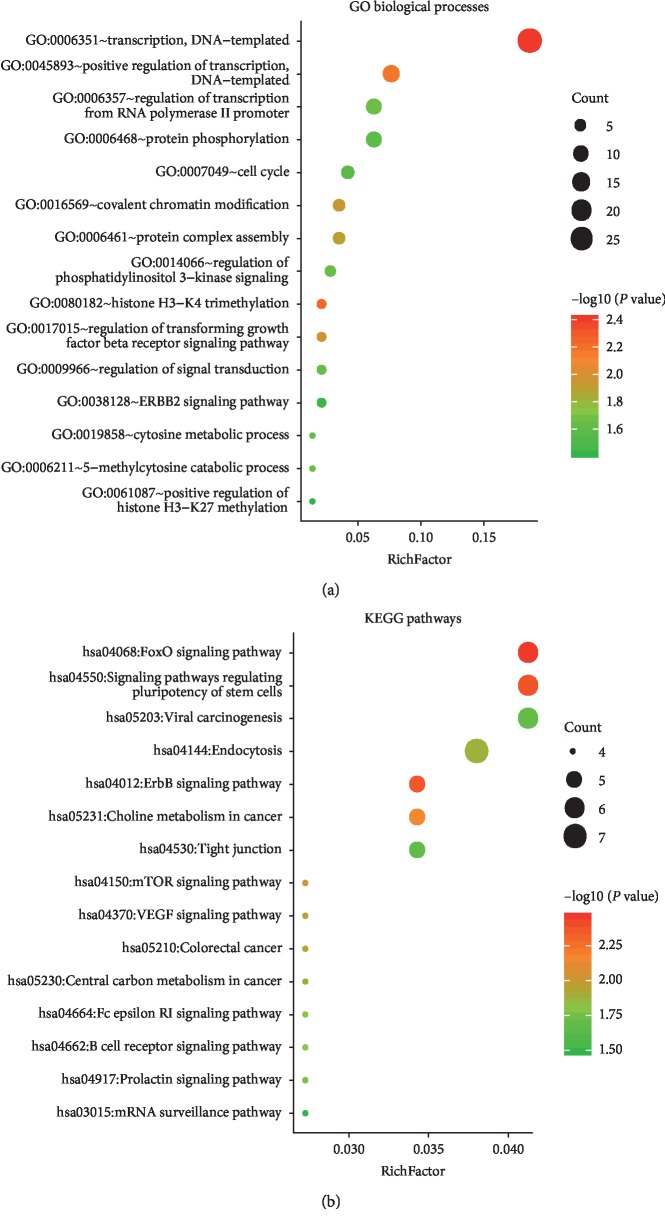
Functional analysis of target genes. (a) Significantly enriched GO biological processes of target genes. (b) Significantly enriched KEGG pathways of target genes. KEGG, Kyoto Encyclopedia of Genes and Genomes; GO, Gene ontology.

The target genes of miR-1301-3p, miR-431-5p, and miR-769-5p were analyzed using four bioinformatics tools: miRDB, TargetScan, miRWalk, and DIANA. We then identified the overlapping target genes for each miRNA obtained from the four online tools using a Venn diagram (Figure 4). As a result, a total of 150 consensus target genes were identified. Moreover, the biological process analysis indicated that, among these genes, there was functional enrichment in chromosome modification, histone remodeling, cell cycle, signal transduction regulation, and transcription regulation. The KEGG pathway results indicated that the genes were significantly enriched in cancer-related pathways, including viral carcinogenesis, forkhead box O (FoxO), vascular endothelial growth factor (VEGF), human epidermal growth factor receptor 2 (ErbB2), and mammalian target of rapamycin (mTOR) signaling pathways (Figure 5).

### 3.5. PPI Network Construction

**Figure 6:**
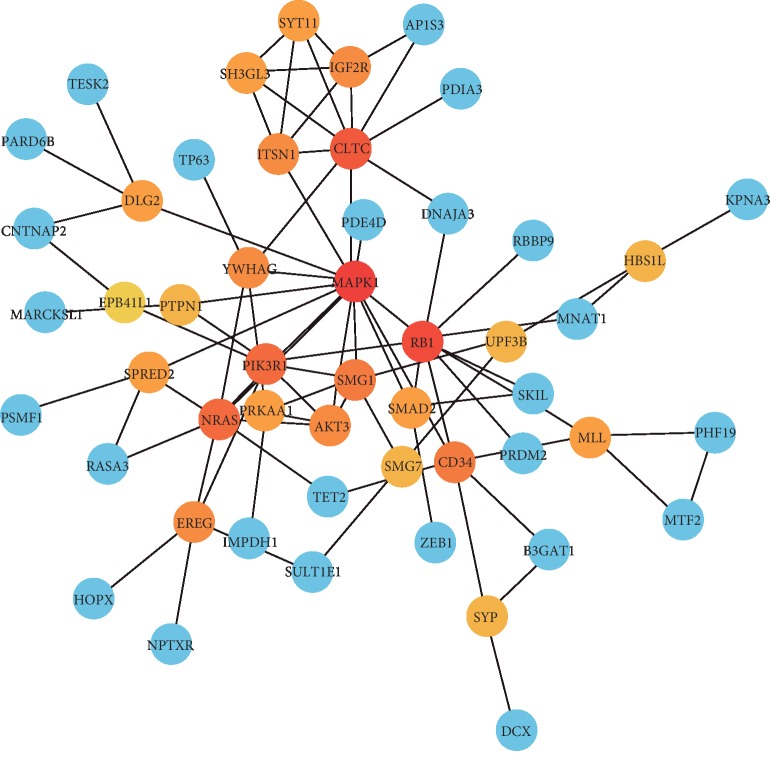
Protein-protein interaction (PPI) network of the target genes. Using the STRING online database, 51 target genes were filtered into the target gene PPI network complex. The three nodes with the highest PPI score are in red.

A PPI network among the target genes of the three miRNAs was established using the STRING database and visualized using Cytoscape (Figure 6). We used “degree more than 6” as the cut-off criterion to identify edge counts of every gene in the PPI network. Of the 150 target genes, 51 were selected to establish the PPI, containing 51 nodes and 84 edges. The three most important hub genes, *MAPK1*, *RB1*, and *CLTC*, were obtained using the Cytoscape plugin CytoNCA.

## 4. Discussion

Due to improvements in screening programs and advancements in endoscopy, the mortality of ESCA patients has decreased over the last several decades. Nevertheless, globally, there were still approximately 509,000 esophageal cancer-related deaths in 2018 [17]. Thus, it is critical to understand the molecular mechanisms of ESCA and explore reliable prognostic biomarkers. A large number of studies have reported that miRNAs could play a crucial role in cancer by functioning as oncogenes or tumor suppressor genes [18]. Moreover, it has been demonstrated that the miRNA profile of tissues exhibits characteristic changes in certain types of tumors [19, 20]. A number of miRNAs have been shown to participate in tumor progression, invasion, and metastasis, including miR-134 [21], miR-145 [22], miR-625 [23], and miR1294 [24]. However, due to clinical heterogeneity, sample insufficiency, and use of various data processing methods, there is inconsistency in the results of these studies.

In this study, we combined two microarray profiling datasets from the GEO database and RNA-seq datasets from the TCGA database to analyze common DEMs in ESCA. A total of 240 normal control tissue samples and 395 ESCA tissue samples were available for the DEM screen, and a total of 33 overlapping DEMs, of which 27 were significantly upregulated and 6 significantly downregulated, were identified. We then determined the prognostic value of each DEM by screening the clinical features downloaded from the TCGA database. Three DEMs, miR-1301-3p, miR-431-5p, and miR-769-5p, were shown to be associated with the OS of ESCA patients. Differential expression analyses of the three DEMs in TCGA validated that they were significantly upregulated in ESCA. It is worth noting that the functions of the three miRNAs identified in our study differ depending on cancer type, and they may act as oncogenic miRNAs or anticancer miRNAs. MiR-431-5p, formerly reported as a particular nervous system miRNA, has also been identified as a novel tumor-related miRNA [25]. It has been shown to be a prognostic biomarker for colorectal cancer (CRC) patients, as it plays an oncogenic role in CRC [26]. However, Rapa et al. have reported that miR-431-5p can inhibit lung carcinoma development, and miR-431-5p is also significantly downregulated in carcinoids that metastasize to the lymph nodes [27]. In addition, miR-769-5p has been involved in the oncogenesis and development of melanoma and can promote the proliferation of this malignancy by suppressing *GSK3B* [28]. However, Zhao et al. demonstrated that miR-769-5p is significantly downregulated in non-small-cell lung carcinoma [29]. As for miR-1301-3p, Song et al. have shown that it promotes prostate cancer by activating the Wnt pathway [30]. Conversely, Chao et al. reported that miR-1301-3p inhibits the migration, invasion, and angiogenesis of hepatocellular carcinoma by targeting BCL9 via Wnt/*β*-catenin signaling [31]. The function of these three miRNAs in ESCA remains poorly understood. Our findings demonstrate that they are upregulated and associated with the prognosis of ESCA.

In order to gain further insight into the molecular function of the three miRNAs, we analyzed their potential target genes, performed functional enrichment analysis, and established the PPI network. The results of GO annotation and KEGG enrichment analysis revealed that the three miRNAs are involved in cancer-related signaling pathways, including the viral carcinogenesis, FoxO, ErbB2, VEGF, and mTOR signaling pathways. It has been reported that FoxO transcription factors regulate tumorigenesis and drug resistance. Maxim et al. showed that the ErbB2/PI3K signaling pathway modifies drug sensitivity in patients with ESCA [32]. VEGF signaling has been demonstrated to play a critical role in the growth and metastasis of multiple cancers [33]. Furthermore, the PI3K/Akt/mTOR signaling pathway can promote cell apoptosis and decrease cell proliferation by reducing Plk1 and could be a potential therapeutic target [34]. In the PPI network, we found three high-degree proteins encoded by the target genes *MAPK1*, *RB1*, and *CLTC*, which play key roles in a variety of tumors. A previous study demonstrated that abnormal expression of *MAPK1* contributes to the occurrence of ESCA [35]. It has been reported that *RB1* expression was downregulated in esophageal small-cell carcinoma [36]. *CLTC* is associated with endocytosis and mitosis in cancer cells [37]. Further functional verification is needed to confirm these predictions, which could provide potential therapeutic targets in the treatment of ESCA.

Our study has several limitations. First, although the identified DEMs were associated with ESCA, no additional experiments were conducted to validate these findings. Second, although we added TCGA data validation to increase our sample size and confirm our findings, the majority of the ESCA samples were from esophageal squamous cell carcinoma. Therefore, further experimental exploration is needed to verify the ability of DEMs to determine the prognosis of ESCA.

## 5. Conclusions

We identified a novel three-miRNA signature (miR-1301-3p, miR-431-5p, and miR-769-5p) as a potential prognostic biomarker for ESCA patients. Further experimental studies with larger sample sizes are needed to validate our findings and elucidate the mechanisms of the three DEMs in esophageal cancer.

## Figures and Tables

**Table 1:** Clinical features of ESCA patients.

Variable	No. of patients (%)
Sex	
Male	145 (86.3)
Female	23 (13.7)

Age (years)	
<60	75 (44.6)
≥60	93 (55.4)

T stage	
T1 + T2	68 (40.5)
T3 + T4	100 (59.5)

Lymph node status	
N0	69 (41.1)
N1–3	85 (50.6)
NA	14 (8.3)

Distant metastasis	
M0	137 (81.5)
M1	16 (9.5)
M*x*	15 (8.9)

Histologic grade	
G*x* + G1	90 (53.6)
G2 + G3	78 (46.4)

Pathologic stage	
I	15 (8.9)
II	76 (45.2)
III	61 (36.3)
IV	16 (9.5)

ESCA: esophageal carcinoma; NA : not available; No: number.

**Table 2:** Association between three miRNAs and clinical features.

Variable	miR-431-5p expression			miR-769-5p expression			miR-1301-3p expression		
	Low	High	*P* value	Low	High	*P* value	Low	High	*P* value
Age, yrs			0.277			0.642			0.003
<60	34	41		36	39		28	47	
≥60	50	43		48	45		56	37	

T Stage			0.342			0.678			0.014
T1	15	8		11	12		18	5	
T2	21	24		26	19		22	23	
T3	45	46		43	48		42	49	
T4	3	6		4	5		2	7	

Lymph node status			0.871			0.399			0.463
N0	35	34		38	31		39	30	
N1–3	42	43		41	44		43	42	

Distant metastasis			0.164			0.320			0. 073
M0	72	65		71	66		73	64	
M1	12	19		13	18		11	20	

Histologic grade			0.757			0.005			0.005
G*x* + G1	46	44		54	36		55	35	
G2 + G3	38	40		30	48		29	49	

Pathologic stage			0.048			0.644			0.105
I	11	4		9	6		11	4	
II	36	40		39	37		40	36	
III	33	28		30	31		28	33	
IV	4	12		6	10		5	11	

Yrs: years.

**Table 3:** Univariate and multivariate analysis in patients with esophageal cancer.

Variable	Univariate analysis			Multivariate analysis		
	HR	95% CI	*P* value	HR	95% CI	*P* value
Age at diagnosis						
>60 vs. ≤60	0.931	0.576–1.506	0.771	0.933	0.567–1.534	0.783
Pathologic stage						
III + IV vs. I + II	2.065	1.530–2.786	<0.001	1.995	1.299–3.064	0.002
T Stage						
T3 + T4 vs. T1 + T2	1.474	1.055–2.060	0.023	1.144	0.813–1.611	0.440
Lymph node status						
N1-2 vs. N0	1.395	1.120–1.738	0.003	1.048	0.805–1.365	0.726
Distant metastasis						
M1 vs. M0	2.163	1.250–3.743	0.006	1.022	0.508–2.057	0.951
Three-miRNA signature						
high-risk vs. low-risk	3.234	1.457–7.174	0.004	3.442	1.475–8.035	0.004

HR, hazard ratio; 95% CI, 95% confidence interval; vs., versus.

## Data Availability

The data used to support the findings of this study are included within the article.

## Supplementary Materials

Supplementary Materials Supplementary Table 1: the names of the common differentially expressed miRNAs (DEMs) among the three datasets. Supplementary Figure 1: Kaplan–Meier survival curves of other 30 differentially expressed miRNA based on TCGA.

## References

[1] Napier K. J., Scheerer M., Misra S. (2014). Esophageal cancer: a review of epidemiology, pathogenesis, staging workup and treatment modalities. *World Journal of Gastrointestinal Oncology*.

[2] Arnal M. J. D., Ferrandez Arenas A., Lanas Arbeloa A. (2015). Esophageal cancer: risk factors, screening and endoscopic treatment in Western and Eastern countries. *World Journal of Gastroenterology*.

[3] Bray F., Ferlay J., Soerjomataram I., Siegel R. L., Torre L. A., Jemal A. (2018). Global cancer statistics 2018: GLOBOCAN estimates of incidence and mortality worldwide for 36 cancers in 185 countries. *CA: A Cancer Journal for Clinicians*.

[4] Pennathur A., Gibson M. K., Jobe B. A., Luketich J. D. (2013). Oesophageal carcinoma. *The Lancet*.

[5] Li Y., Liu Y., Fan J. (2018). Validation and bioinformatic analysis of propofol-induced differentially expressed microRNAs in primary cultured neural stem cells. *Gene*.

[6] Griffiths-Jones S., Grocock R. J., van Dongen S., Bateman A. (2006). miRBase: microRNA sequences, targets and gene nomenclature. *Nucleic Acids Research*.

[7] Tan C., Qian X., Guan Z. (2016). Potential biomarkers for esophageal cancer. *SpringerPlus*.

[8] Hong L., Han Y., Zhang H., Zhao Q., Wu K., Fan D. (2014). Prognosis-related microRNAs in esophageal cancer. *Expert Opinion on Biological Therapy*.

[9] Guo L., Zheng L., Zhao Y., Wang Q. (2018). Profiling and bioinformatic analyses indicate differential circRNA and miRNA/isomiR expression and interactions. *BioMed Research International*.

[10] Chen Z., Li J., Tian L. (2014). MiRNA expression profile reveals a prognostic signature for esophageal squamous cell carcinoma. *Cancer Letters*.

[11] Barrett T., Wilhite S. E., Ledoux P. (2013). NCBI GEO: archive for functional genomics data sets-update. *Nucleic Acids Research*.

[12] Chandran U. R., Medvedeva O. P., Barmada M. M. (2016). TCGA expedition: a data acquisition and management system for TCGA data. *PLoS One*.

[13] Robinson M. D., McCarthy D. J., Smyth G. K. (2010). edgeR: a Bioconductor package for differential expression analysis of digital gene expression data. *Bioinformatics*.

[14] McCarthy D. J., Chen Y., Smyth G. K. (2012). Differential expression analysis of multifactor RNA-Seq experiments with respect to biological variation. *Nucleic Acids Research*.

[15] Huang D. W., Sherman B. T., Lempicki R. A. (2009). Systematic and integrative analysis of large gene lists using DAVID bioinformatics resources. *Nature Protocols*.

[16] Tang Y., Li M., Wang J., Pan Y., Wu F.-X. (2015). CytoNCA: a cytoscape plugin for centrality analysis and evaluation of protein interaction networks. *Biosystems*.

[17] Sakamoto T., Fujiogi M., Matsui H., Fushimi K., Yasunaga H. (2019). Comparing perioperative mortality and morbidity of minimally invasive esophagectomy versus open esophagectomy for esophageal cancer. *Annals of Surgery*.

[18] Kim D., Chang H. R., Baek D. (2017). Rules for functional microRNA targeting. *BMB Reports*.

[19] Mari L., Hoefnagel S. J. M., Zito D. (2018). microRNA 125a regulates MHC-I expression on esophageal adenocarcinoma cells, associated with suppression of antitumor immune response and poor outcomes of patients. *Gastroenterology*.

[20] Jiao W., Zhang J., Wei Y. (2019). MiR-139-5p regulates VEGFR and downstream signaling pathways to inhibit the development of esophageal cancer. *Digestive and Liver Disease*.

[21] Klimczak-Bitner A. A., Kordek R., Bitner J., Musiał J., Szemraj J. (2016). Expression of MMP9, SERPINE1 and miR-134 as prognostic factors in esophageal cancer. *Oncology Letters*.

[22] Han Q., Zhang H.-Y., Zhong B.-L., Wang X.-J., Zhang B., Chen H. (2016). MicroRNA-145 inhibits cell migration and invasion and regulates epithelial-mesenchymal transition (EMT) by targeting connective tissue growth factor (CTGF) in esophageal squamous cell carcinoma. *Medical Science Monitor*.

[23] Li C., Li D. C., Che S. S. (2015). The decreased expression of miR-625 predicts poor prognosis of esophageal squamous cell carcinoma. *International Journal of Clinical and Experimental Medicine*.

[24] Liu K., Li L., Rusidanmu A., Wang Y., Lv X. (2015). Down-regulation of MiR-1294 is related to dismal prognosis of patients with esophageal squamous cell carcinoma through elevating C-myc expression. *Cellular Physiology and Biochemistry*.

[25] Sun K., Zeng T., Huang D. (2015). MicroRNA-431 inhibits migration and invasion of hepatocellular carcinoma cells by targeting the ZEB1-mediated epithelial-mensenchymal transition. *FEBS Open Bio*.

[26] Kanaan Z., Roberts H., Eichenberger M. R. (2013). A plasma MicroRNA panel for detection of colorectal adenomas. *Annals of Surgery*.

[27] Rapa I., Votta A., Felice B. (2015). Identification of microRNAs differentially expressed in lung carcinoid subtypes and progression. *Neuroendocrinology*.

[28] Qiu H.-j., Lu X.-h., Yang S.-s., Weng C.-y., Zhang E.-k., Chen F.-c. (2016). MiR-769 promoted cell proliferation in human melanoma by suppressing GSK3B expression. *Biomedicine & Pharmacotherapy*.

[29] Yang Z., He J., Gao P. (2017). miR-769-5p suppressed cell proliferation, migration and invasion by targeting TGFBR1 in non-small cell lung carcinoma. *Oncotarget*.

[30] Song X.-L., Huang B., Zhou B.-W. (2018). miR-1301-3p promotes prostate cancer stem cell expansion by targeting SFRP1 and GSK3*β*. *Biomedicine & Pharmacotherapy*.

[31] Yang C., Xu Y., Cheng F. (2017). miR-1301 inhibits hepatocellular carcinoma cell migration, invasion, and angiogenesis by decreasing Wnt/*β*-catenin signaling through targeting BCL9. *Cell Death & Disease*.

[32] Issaenko O. A., Bitterman P. B., Polunovsky V. A., Dahlberg P. S. (2012). Cap-dependent mRNA translation and the ubiquitin-proteasome system cooperate to promote ERBB2-dependent esophageal cancer phenotype. *Cancer Gene Therapy*.

[33] Xu X.-L., Ling Z.-Q., Chen W., Xu Y.-P., Mao W.-M. (2013). The overexpression of VEGF in esophageal cancer is associated with a more advanced TMN stage: a meta-analysis. *Cancer Biomarkers*.

[34] Tasioudi K. E., Sakellariou S., Levidou G. (2015). Immunohistochemical and molecular analysis of PI3K/AKT/mTOR pathway in esophageal carcinoma. *APMIS*.

[35] Wang X., Zheng Y., Fan Q., Zhang X. (2013). Effect of blocking Ras signaling pathway with K-Ras siRNA on apoptosis in esophageal squamous carcinoma cells. *Journal of Traditional Chinese Medicine*.

[36] Ishida H., Kasajima A., Kamei T. (2017). SOX2 and Rb1 in esophageal small-cell carcinoma: their possible involvement in pathogenesis. *Modern Pathology*.

[37] Bond M. J., Bleiler M., Harrison L. E. (2018). Spindle assembly disruption and cancer cell apoptosis with a CLTC-binding compound. *Molecular Cancer Research*.

