# Supplementary material for: A Novel Three-miRNA Signature Identified Using Bioinformatics Predicts Survival in Esophageal Carcinoma
Source: Biomed Res Int. 2020 Feb 10;2020:5973082. doi: 10.1155/2020/5973082 (PMC7035545; doi:10.1155/2020/5973082)
Supplement: Supplementary Materials — Supplementary Table 1: the names of the common differentially expressed miRNAs (DEMs) among the three datasets. Supplementary Figure 1: Kaplan–Meier survival curves of other 30 differentially expressed miRNA based on TCGA. [file 5973082.f1.pdf]

Supplementary Table 1. The names of the common differentially expressed miRNAs (DEMs) among the three datasets

DEMs	
Up-regulated	miR-181a, miR-183-3p, miR-196a, miR−218, miR−23b, miR−34c-5p, miR−370, miR−409-5p, miR-431-5p, miR−492, miR−493, miR−503, miR−523, miR−542-5p, miR−548a−3p, miR−574, miR−631, miR−663b, miR−720, miR-767, miR-769-5p, miR−787, miR−796, miR−870, miR-7-5p, miR-1180, miR-1301-3p,
Down-regulated	miR−1, miR−139-5p, miR−150, miR−375,miR−497, miR−1224-5p

The cut-off criteria is $P < 0.05$ and $|log2FC| > 1.0$.

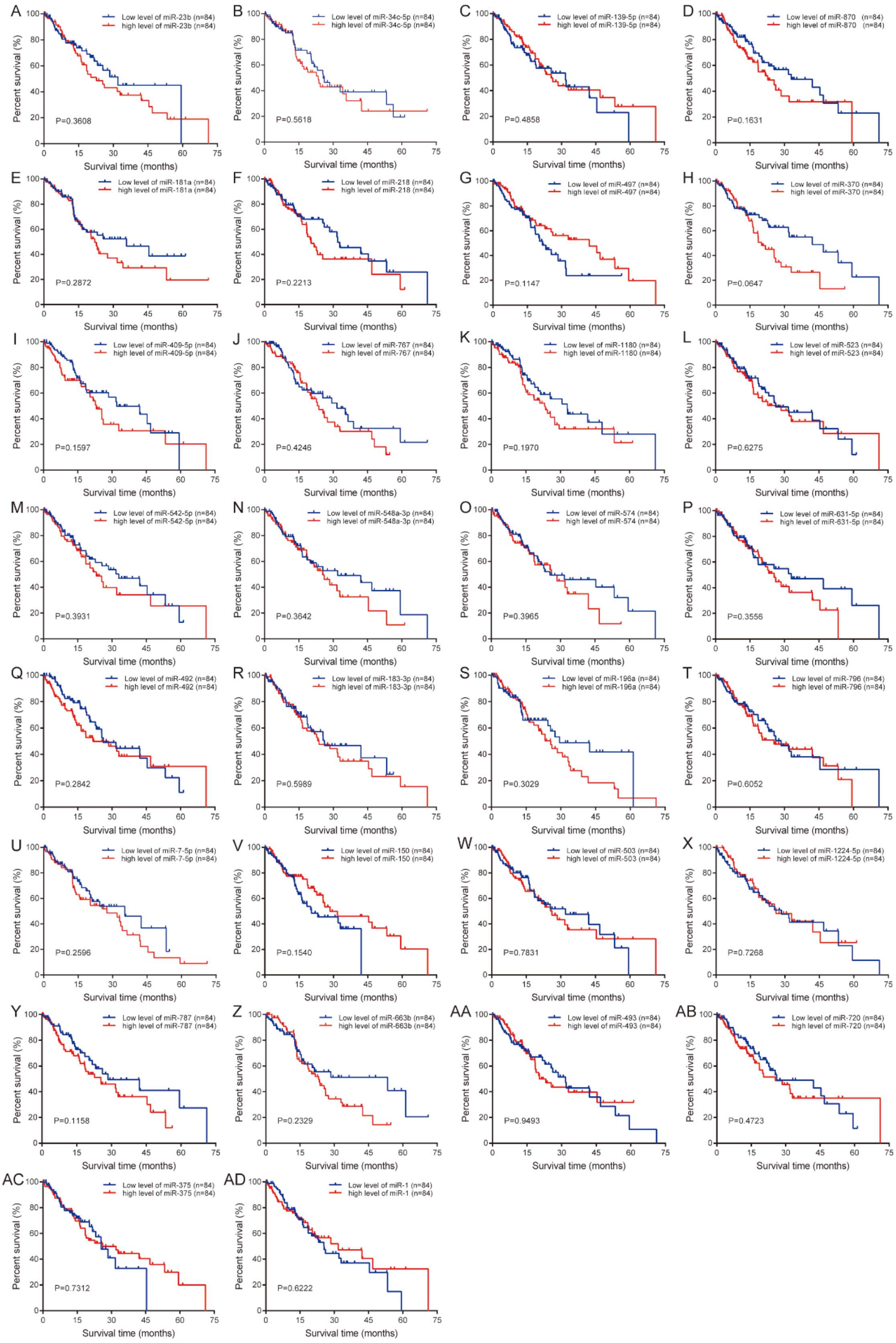

Supplementary Figure 1. Kaplan-Meier survival curves of other 30 differentially expressed miRNA based on TCGA
